# Enhancer-bound Nrf2 licenses HIF-1α transcription under hypoxia to promote cisplatin resistance in hepatocellular carcinoma cells

**DOI:** 10.18632/aging.202137

**Published:** 2020-12-03

**Authors:** Xin Jin, Liansheng Gong, Ying Peng, Le Li, Gang Liu

**Affiliations:** 1 Department of Nuclear Medicine, Key Laboratory of Nanobiological Technology of Chinese Ministry of Health, Xiangya Hospital, Central South University, Changsha 410008, Hunan, China; 2 Department of Biliary Surgery, Xiangya Hospital, Central South University. Changsha 410008, Hunan, China; 3 Department of International Joint Research Center of Minimally Invasive Endoscopic Technology Equipment and Standards, Xiangya Hospital, Central South University, Changsha 410008, Hunan, China; 4 Hunan Yuantai Biotechnology Co., Ltd, Changsha 410000, Hunan, China

**Keywords:** tumor microenvironment, hypoxia, chemoresistance, Nrf2, HIF-1α

## Abstract

Tumor microenvironment is hypoxic, which can cause resistance to chemotherapy, but the detailed mechanisms remain elusive. Here we find that mild hypoxia (5% O_2_) further increases cisplatin resistance in the already resistant HepG2/DDP but not the sensitive HepG2 cells. We find that Nrf2 is responsible for cisplatin resistance under hypoxia, as Nrf2 knockdown sensitizes HepG2/DDP cells while Nrf2 hyper-activation (though KEAP1 knockdown) increases resistance of HepG2 cells to cisplatin. Nrf2 binds to an enhancer element in the upstream of HIF-1α gene independently of hypoxia, promoting HIF-1α mRNA synthesis under hypoxic condition. As a result, Nrf2-dependent transcription counteracts HIF-1α degradation under mild hypoxia condition, leading to preferential cisplatin-resistance in HepG2/DDP cells. Our data suggest that Nrf2 regulation of HIF-1α could be an important mechanism for chemotherapy resistance *in vivo*.

## INTRODUCTION

In most solid tumors, abnormal proliferation of cancer cells rapidly overwhelms angiogenesis, leading to leakiness and compression of blood vessels, and leaving large areas of tumor with insufficient blood supply. As a result, a differential hypoxic microenvironment is formed inside the tumors [1, 2]. Lack of blood flow forms a physical barrier that prevents effective drug delivery [3]. In addition, the hypoxic environment can modulate complex metabolic pathways, therefore promoting tumor proliferation, metastasis and resistance to treatments such as chemotherapy, radiotherapy and immunotherapy [4–7].

The hypoxia inducible factor-1 (HIF-1) plays an important role in liver cancer [8, 9]. HIF-1 is a hypoxia-inducible heterodimeric transcription factor composed of HIF-1α and HIF-1β subunits [10, 11]. Under normal condition, the HIF-1α protein is continuously degraded through proteasome mediated by VHL-dependent ubiquitination [12]. Upon hypoxic stress, however, VHL is inhibited, allowing HIF-1α to enter cell nucleus to activate hypoxia-inducible gene transcription [13]. After forming a heterodimer with HIF-1β on target gene promoters, HIF-1 drives the transcription required for adaptation to the hypoxic condition [11, 14].

Cisplatin is widely used to treat a broad range of cancers [15, 16]. The molecular mechanisms of cisplatin cytotoxicity involve DNA crosslinks, which causes DNA damage and interference with DNA replication [17]. However, cisplatin treatment has been well documented to cause drug resistance [18]. For example, hypoxia can induce ROS production and facilitates mitochondrial fission, leading to cisplatin resistance in ovarian cancer cells [19]. Hypoxia exposure can also induce cisplatin resistance via signaling pathways involving p53, HIF-1α, SIRT1, AMPK and autophagy etc. [20–24].

NF-E2-related factor 2 (NRF2) is also involved in cisplatin resistance in multiple cancer types [25–29]. Nrf2 is a leucine zipper (bZip) transcription factor required for the oxidative homeostasis. In response to reactive oxygen species (ROS) and other electrophiles, Nrf2 activates gene transcription of a set of drug-metabolizing enzymes, such as glutathione S-transferase (GST) and NAD(P)H: quinone oxidoreductase 1 (NQO1) [30, 31]. Nrf2 protein normally is sequestered in the cytoplasm by Kelch-like ECH-associated protein 1 (KEAP1), which promotes Nrf2 degradation through proteasome [32–34]. Upon oxidative stress, Nrf2 dissociates from KEAP1, accumulates in the nucleus and binds to antioxidant response element (ARE) in the promoter/enhancer of its target genes [34]. *KEAP1* or *NRF2* genes are often mutated in cancer cells [35–38].

How hypoxia modulates the tumor microenvironment and confers growth advantages to cancer cells is currently a key question in cancer research. Especially, in-depth knowledge remains scarce regarding the molecular mechanisms of hypoxia-induced cisplatin resistance. In this study, we investigate the cisplatin sensitivity of hepatocellular carcinoma cell HepG2 under hypoxia conditions. We find that mild hypoxia (5% O_2_) further increases cisplatin resistance in the already resistant HepG2/DDP cells but not the sensitive HepG2 cells. Further studies show that deregulation of Nrf2 in HepG2/DDP cells enhances HIF-1α expression by directly binding to an enhancer element in the upstream of HIF-1α gene, which contributes to the cisplatin resistance under hypoxia conditions. Our study demonstrates a direct role of Nrf2 in regulation of hypoxia-induced chemo-resistance in liver cancer cells, providing better understanding of how tumor microenvironment affects chemotherapy.

## RESULTS

### Differential HIF-1α induction and cisplatin resistance in HepG2/DDP versus HepG2 cells

**Figure 1:**
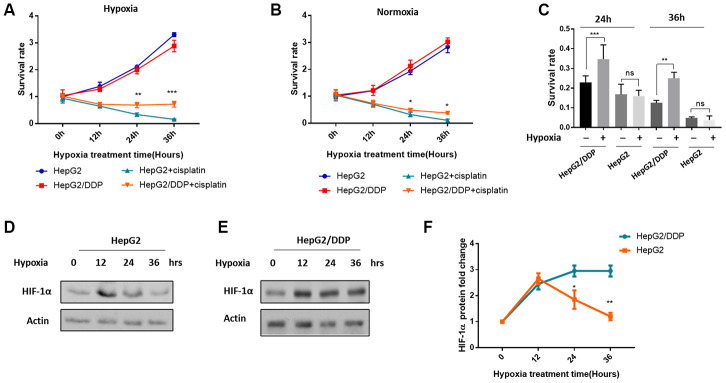
**Differential HIF-1α induction and cisplatin resistance in HepG2/DDP versus HepG2 cells under mild hypoxia.** (**A**) Cisplatin-mediated cytotoxicity in hepatocellular carcinoma cells under mild hypoxic condition (5% O_2_). The cisplatin-resistant (HepG2/DDP) and cisplatin-sensitive (HepG2) cells at 60% confluency were treated with or without 10 ug/ml cisplatin and incubated at mild hypoxic condition (5% O_2_). Cells were collected at indicated time points and analyzed by using CellTiter-Glo luminescence cell viability assay kit. Data from multiple independent experiments were normalized to the value at time 0. Student’s t-test was performed to evaluate the statistical significance. **P<0.001, ***P<0.0001. (**B**) Cisplatin-mediated toxicity in hepatocellular carcinoma cells under normoxia (21% O_2_). Experiments were performed as in (**A**) except that cells were incubated in the regular incubator with at 21% oxygen. Data were from multiple independent experiments and normalized to the value at time 0. Student’s t-test: *P<0.01. (**C**) Mild hypoxia (5% O_2_) induced cisplatin resistance in HepG2/DDP but not HepG2 cells. Data from both (**A**) and (**B**) after 24h and 36h of cisplatin treatment under normoxia or hypoxia were normalized to none-treated controls. Student’s t-test: ns, not significant, **P<0.001, ***P<0.0001. (**D**) Mild hypoxia (5% O_2_) transiently increased HIF-1α protein levels in HepG2 cells. HepG2 cells at 60% confluency were incubated at 5% O_2_ for indicated time point. HIF-1α protein levels were examined by Western blot using specific antibody against HIF-1α and Actin control. (**E**) Mild hypoxia (5% O_2_) caused sustained elevation of HIF-1α protein in HepG2/DDP cells. Experiments were performed as in (**D**) except HepG2/DDP cells were used. (**F**) Comparison of induction of HIF-1α under mild hypoxic condition (5% O_2_) in HepG2 and HepG2/DDP cells. Several repeats of Western blot in (**D**) and (**E**) were quantified and normalized to time 0. Student’s t-test: *P<0.01, **P<0.001.

We were initially aiming at understanding how hypoxia would impact chemotherapy results. To this end, we cultured the hepatocellular carcinoma cell lines HepG2 (cisplatin-sensitive) and HepG2/DDP (cisplatin-resistant) under normal and mild hypoxic condition (5% O_2_), and at the same time treated with or without 10 ug/ml cisplatin. Such a mild hypoxia did not obviously prevent the growth of cancer cells (Figure 1A, 1B). We then examined the survival of cisplatin-treated and untreated cells in a time course of 0, 12, 24 and 36 hours. As expected, HepG2/DDP cells were more resistant to cisplatin at both normoxia and hypoxia conditions (Figure 1A, 1B). Interestingly, we noticed that under hypoxia, the difference between HepG2 and HepG2/DDP cells was more pronounced (compare Figure 1A, 1B). We then compared the ability of mild hypoxia (5% O_2_) to increase cisplatin resistance in HepG2 and HepG2/DDP cells. Our data showed that, importantly, such mild hypoxic stress only further increased cisplatin resistance in HepG2/DDP cells but not in HepG2 cells (Figure 1C).

Since hypoxia is known to increase HIF-1α protein levels and induce transcription programs leading to chemo-resistance, we hypothesized that HIF-1α might be activated in a different manner in HepG2 and HepG2/DDP cells. To test this possibility, we examined the HIF-1α expression and induction under 10 ug/ml cisplatin treatment. Consistent with previous study, hypoxia increased HIF-1α protein levels shortly after 12 hours in both HepG2 and HepG2/DDP cells. Intriguingly, we found that HIF-1α was only transiently induced in HepG2 cells: HIF-1α protein levels began to decrease at 24 hours and reached the basal level at 36 hours (Figure 1D, 1F). However, in HepG2/DDP cells, HIF-1α protein remained stably expressed during the 36-hour hypoxic stress (Figure 1E, 1F). Together, our studies show that compared with HepG2, HepG2/DDP cells are not only more resistant to cisplatin, but also better responsive to hypoxic induction of cisplatin-resistance and HIF-1α expression.

### Nrf2 activation is required for sustained HIF-1α induction in HepG2/DDP cells

**Figure 2:**
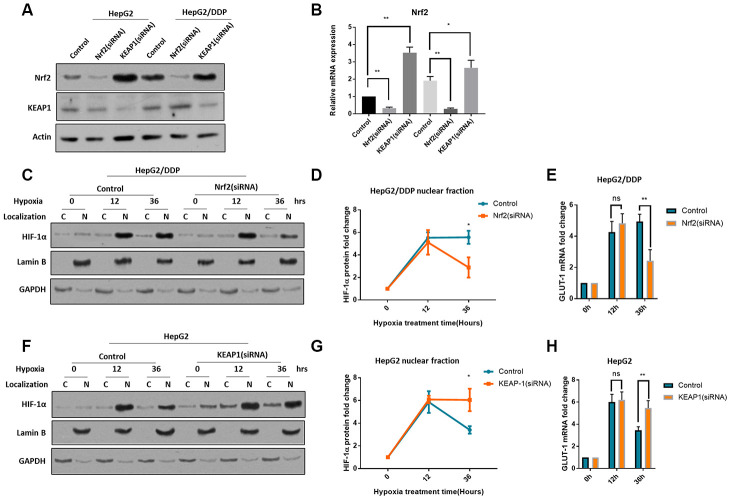
**Nrf2 hyper-activation is required for sustained HIF-1α induction in HepG2/DDP cells.** (**A**) Up-regulation of Nrf2 by KEAP1 knockdown. HepG2 and HepG2/DDP cells were transfected with siRNA specific to Nrf2 or KEAP1. Protein levels of Nrf2, KEAP1 and actin control were examined by Western blot. (**B**) Quantification of 3 experiments in (**A**). Student’s t-test was performed to evaluate the statistical significance. *P<0.01, **P<0.001. (**C**) Nrf2 knockdown blocked the sustained induction of HIF-1α in HepG2/DDP cells by hypoxia. Cells were transfected with Nrf2-specific siRNA for 24 hours, then incubated at hypoxic condition (5% O_2_) for indicated time. Nuclear (N) and cytoplasm (**C**) were separated. HIF-1α, Lamin B (marker for nucleus) and GAPDH (marker for cytoplasm) were examined by Western blot using specific antibodies. (**D**) Quantification of nuclear HIF-1α protein levels in HepG2/DDP cells from 2 experiments shown in (**C**). Data were normalized to time 0. Student’s t-test: *P<0.01. (**E**) Nrf2 knockdown blocked the sustained induction of HIF-1α target gene *GLUT-1* in HepG2/DDP cells by hypoxia. Cells were transfected with Nrf2-specific siRNA for 24 hours, then incubated at hypoxic condition (5% O_2_) for indicated time. Total mRNA was extracted and reversed transcribed. *GLUT-1* cDNA was examined by RT-qPCR. Data from 3 experiments were plotted and analyzed with Student’s t-test: ns, not significant, **P<0.001. (**F**) KEAP-1 knockdown blocked the rapid degradation of HIF-1α in HepG2 under hypxia. siRNA knockdown, nuclear purification and Western blot was performed as shown in (**C**). (**G**) Quantification of Western blot signals in (**F**). Data were normalized to time 0. Student’s t-test was performed to evaluate the statistical significance. *P<0.01. (**H**) KEAP1 knockdown blocked *GLUT-1* down-regulation in HepG2 cells under hypoxia. Data from 2 repeats normalized to time 0. Student’s t-test: *P<0.01, **P<0.001.

To understand how HIF-1α was differently regulated in HepG2 and HepG2/DDP cells, we checked Nrf2 pathway. Nrf2 pathway has recently been implicated in chemo-resistance in many cancers [25–29]. The cisplatin-resistant HepG2/DDP cells were derived from HepG2 cells by continuously culturing in the presence of cisplatin. One of the mechanisms that contributes to the cisplatin-resistant is the hyperactivation of Nrf2 [39]. To check if Nrf2 was involved, we first decreased or increased Nrf2 expression by siRNA of Nrf2 or KEAP1, respectively. Nrf2 baseline expression was higher in HepG2/DDP cells but was decreased by Nrf2 siRNA to the same levels in HepG2 cells; KEAP1 knockdown can robustly increase Nrf2 protein levels in both cells (Figure 2A, 2B), confirming the successful effect of siRNA knockdowns.

We then stressed cells with hypoxia (5% O_2_) for 36 hours. We separated the nuclei and cytoplasm and examined HIF-1α protein levels by western blot. The results showed that, HIF-1α was mostly present in the nucleus (Figure 1C, 1F), consistently with previous reports [40, 41]. Interestingly, lack of Nrf2 did not affect the HIF-1α induction in in HepG2/DDP cells, however, after 36-hour hypoxia, Nrf2 knockdown significantly reduced the HIF-1α protein levels (Figure 2C, 2D). Consistently, the mRNA levels of GLUT-1, a HIF-1α target gene [42], were changed similarly (Figure 2E). In HepG2 cells, Nrf2 activation by KEAP1 knockdown did not affect the induction of HIF-1α, however, Nrf2 activation blocked the rapid degradation of HIF-1α after 36 hours of hypoxia (Figure 2F, 2G). Consistently, the mRNA levels of target gene GLUT-1 were changed similarly (Figure 2H). In a way, Nrf2 modulation switched the induction pattern of HepG2 and HepG2/DDP cells (compare 2D to 2G). Together, these results reveal an important role of Nrf2 in the regulation of HIF-1α protein levels under hypoxia and suggest a novel mechanism for cisplatin-resistance in the tumor micro-environment.

### Nrf2 promotes hypoxia-induced cisplatin resistance in HepG2/DDP cells through HIF-1α

**Figure 3:**
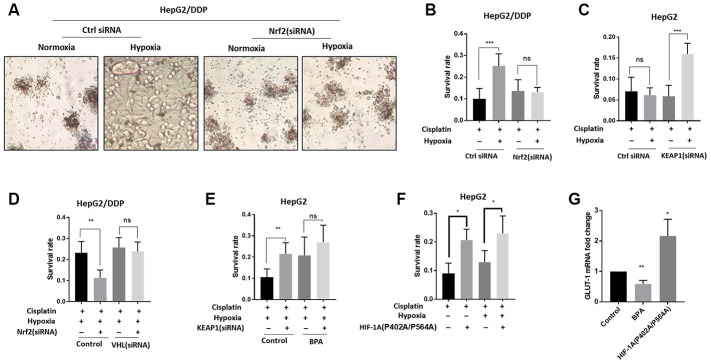
**Nrf2 promotes hypoxia-induced cisplatin resistance in HepG2/DDP cells through HIF-1α.** (**A**) Nrf2 knockdown blocked the hypoxia-induced cisplatin-resistance in HepG2/DDP cells. Cells were transfected with Nrf2-specific siRNA for 24 hours, then incubated under normoxic (21% O_2_) or hypoxic conditions (5% O_2_) for 36 hours in the presence of 10ug/ml cisplatin. Cells were imaged in the 96-well plate. Representative images were shown. (**B**) CellTiter-Glo viability assay for cells shown in (**A**). HepG2/DDP cells treated with cisplatin for 36 hours or non-treated controls were directly lyzed with CellTiter-Glo reagent and read by luminescence reader. Data collected from 3 biological repeats were normalized to non-treated control. Student’s t-test was performed to evaluate the statistical significance. ns, not significant, ***P<0.0001. (**C**) Nrf2 activation by KEAP1 knockdown promoted hypoxia-induced cisplatin resistance in HepG2 cells. KEAP1 was knocked down as in (**A**) and cell viability was measured as in (**B**). Student’s t-test: ns, not significant, ***P<0.0001. (**D**) HIF-1α activation prevented Nrf2 knockdown from sensitizing HepG2/DDP cells to cisplatin. Cells were transfected with indicated siRNA and treated with 5% O_2_ as in (A). CellTiter-Glo viability assay was conducted as in (**B**) for 3 biological replicates. Student’s t-test: **P<0.001, ns, not significant. (**E**) HIF-1α inhibition prevented KEAP1 knockdown from increasing cisplatin resistance in HepG2 cells. HIF-1α was inhibited with an established chemical inhibitor dimethyl-bisphenol A (BPA). HepG2 cells with or without KEAP1 knockdown were treated with cisplatin and BPA as indicated and incubated under normoxia or hypoxia for 36 hours. CellTiter-Glo viability assay was performed as in (**B**) for 3 biological replicates. Student’s t-test: **P<0.001, ns, not significant. 100uM BPA increased cisplatin resistance for unknown reasons. (**F**) Expression of constitutively active HIF-1α increased cisplatin resistance in HepG2 cells. Cells were transfected with plasmid expressing HIF-1α (P402A/P564A) for 24 hours then treated with cisplatin and hypoxia as indicated for 36 hours. Viability was measured by CellTiter-Glo. Student’s t-test: **P<0.001, ns, not significant. (**G**) HIF-1α transcriptional activity was down-regulated and upregulated by BPA and HIF-1α (P402A/P564A), respectively. HepG2 cells were treated with BPA or transfected with HIF-1α (P402A/P564A). Total mRNA was extracted and reversed transcribed. *GLUT-1* cDNA was examined by RT-qPCR. Data from 3 experiments were plotted and analyzed with Student’s t-test: *P<0.01, **P<0.001.

Next, we tested if Nrf2 regulation of HIF-1α could contribute to the increased cisplatin resistance under hypoxia conditions. We first knocked down Nrf2 expression in HepG2/DDP cells and examined cisplatin cytotoxicity under normoxia or 36-hour hypoxia. Significantly, Nrf2 knockdown prevented hypoxia-induced cisplatin resistance in HepG2/DDP cells (Figure 3A, 3B). Second, we up-regulated Nrf2 protein levels through siRNA knocking down of KEAP1 in HepG2 cells. Consistently, Nrf2 hyper-activation increased cisplatin resistance under hypoxic condition (Figure 3C).

We further asked if the Nrf2-dependent cisplatin resistance in HepG2/DDP cells relied on HIF-1α. For this reason, we genetically and pharmacologically modulated the activity of both Nrf2 and HIF-1α. As shown in Figure 3B, the hypoxia-induced cisplatin resistance in HepG2/DDP cells could be reduced by Nrf2 siRNA (Figure 3D, control), however, such reduction was prevented when HIF-1α was activated by knocking down of its negative regulator VHL (Figure 3D, VHL siRNA). In HepG2 cells, KEAP1 knockdown increased cisplatin resistance, but did not when HIF-1α was inhibited by a small molecule inhibitor, Dimethyl-bisphenol A (BPA) (Figure 3E). These results support a model in which Nrf2 increases HIF-1α protein levels in the hypoxic tumor micro-environment to confer cisplatin resistance. For unknown reasons, 100uM BPA increase cisplatin resistance (Figure 3E).

Finally, we overexpressed constitutively active HIF-1α in HepG2 cells to test if it would confer cisplatin resistance. The HIF-1α (P402A/P564A) is constitutively active due to its nondegradable feature [43]. Confirming the hypothesis, HIF-1α (P402A/P564A) overexpression was sufficient to confer cisplatin resistance to HepG2 cells in both normoxia and hypoxia conditions (Figure 3F). As controls, we confirmed that BPA treatment and HIF-1α (P402A/P564A) overexpression decreased and increased, respectively, the transcriptional activity of HIF-1α, as judged from the mRNA expression of HIF-1α target gene *GLUT-1* (Figure 3G).

### Nrf2 binds to an enhancer element of *HIF-1A* in a hypoxia-insensitive manner.

**Figure 4:**
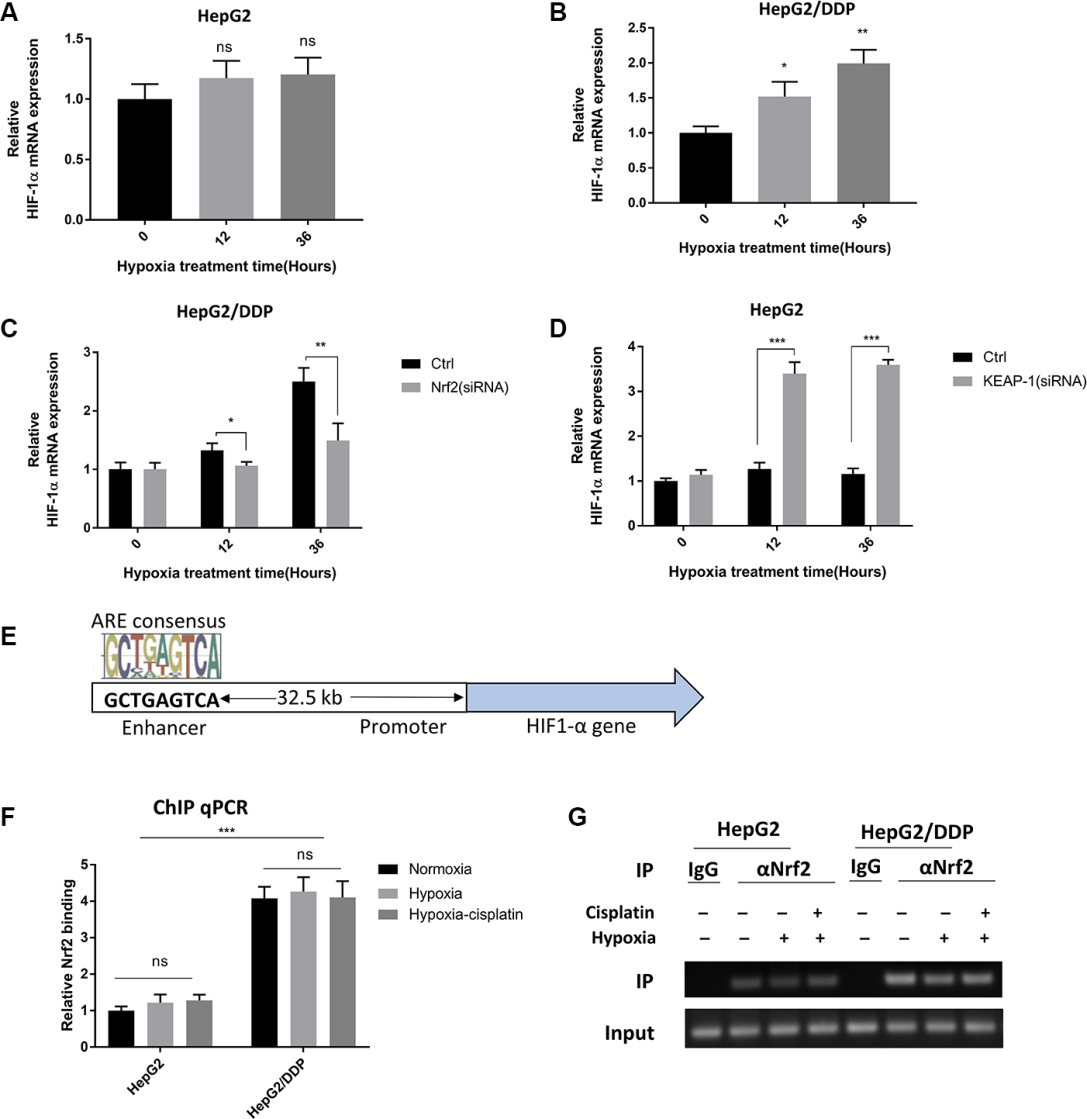
**Nrf2 binds to a *HIF-1A* enhancer element in a hypoxia-insensitive manner.** (**A**) HIF-1α mRNA levels were not changed by hypoxia in HepG2 cells. HepG2 cells were incubated under hypoxic condition (5% O_2_) for indicated time. mRNA was prepared and RT-qPCR was performed with *HIF-1A* specific primers. Student’s t-test was performed to evaluate the statistical significance: ns, not significant. (**B**) HIF-1α mRNA was up-regulated by hypoxia in HepG2 cells. Experiments were performed as in (**A**). Student’s t-test was performed to evaluate the statistical significance. *P<0.01, **P<0.001. (**C**) Hypoxia-induced HIF-1α mRNA expression was Nrf2-dependent in HepG2/DDP cells. HepG2/DDP cells were transfected with Nrf2-specific siRNA then incubated under mild hypoxic condition (5% O_2_) for indicated time. RT-qPCR was performed as in (**A**). Student’s t-test: *P<0.01, **P<0.001. (**D**) Nrf2 hyper-activation increased HIF-1α mRNA expression in HepG2 cells under hypoxia. HepG2 cells were transfected with KEAP1-specific siRNA then incubated with 5% O_2_ for indicated time points. RT-qPCR was performed as in (**A**). Student’s t-test: ***P<0.0001. (**E**) Diagram showing the conserved Nrf2 binding site at the 5’-end of HIF-1α gene. This site has been shown to be bound by Nrf2 and regulate HIF-1α expression before. (**F**) Nrf2 binding to HIF-1α enhancer stronger in HepG2/DDP than HepG2 cells and was not sensitive to hypoxia. HepG2 and HepG2/DDP cells were chromatin-immuno-precipitated (ChIP) by using Nrf2-specific antibody. The binding of the conserved site shown in (**E**) was detected by RT-qPCR. Student’s t-test: ***P<0.0001, ns, not significant. (**G**) Confirming Nrf2 binding to HIF-1α enhancer by regular PCR. Indicated ChIP samples from (**F**) were amplified with specific primers to the conserved Nrf2 binding site by regular PCR then subject to DNA electrophoresis.

We were intrigued to know in detail how Nrf2 regulates HIF-1α protein expression under hypoxia conditions. Since Nrf2 is a transcription factor, we tested if Nrf2 increased HIF-1α gene transcription under hypoxia conditions. We measured the mRNA abundance by RT-qPCR and found that the HIF-1α mRNA levels were elevated in HepG2/DDP cells but not HepG2 cells after hypoxic stress (Figure 4A, 4B). We knocked down Nrf2 by siRNA and asked if increased HIF-1α expression was Nrf2-dependent. As a result, Nrf2.siRNA significantly attenuated the mRNA expression of HIF-1α under hypoxia in HepG2/DDP cells (Figure 4C). Consistently, hyper-activation of Nrf2 pathway in HepG2 cells robustly enhanced the hypoxic induction of HIF-1α expression (Figure 4D). These results indicate that Nrf2 regulated HIF-1α at the transcriptional levels.

We next asked if Nrf2 could directly target HIF-1α gene for regulation. By using genome-wide ChIP-seq analysis, a recent report [44] shows that Nrf2 binds to an enhancer at the 5’-end of *HIF1A* gene to modulate its expression, and there is no other conserved Nrf2 binding sites on HIF-1A promoter (Figure 4E). We tested if Nrf2 regulation of HIF-1α mRNA levels in HepG2/DDP cells could be attributed to this enhancer. By Nrf2 ChIP analysis coupled with RT-qPCR, our experiment showed that indeed, Nrf2 was bound to the HIF-1α enhancer element in HepG2/DDP cells (Figure 4F, 4G). Interestingly, such binding was insensitive to hypoxia. In HepG2 cells, weak association was found and was also hypoxia-insensitive (Figure 4F, 4G). These results suggest that Nrf2 functions to enhance hypoxia-induce drug resistance through direct regulation of HIF-1α gene transcription.

## DISCUSSION

A major hurdle to successful cancer treatment is the development of drug resistance in cancer cells [45]. Drug resistance, also referred to as chemo-resistance, could be developed by intrinsic genetic mutations or by environmental factor such as extra-cellular signaling [45]. Hypoxia (due to insufficient oxygen supplies) is one of such environmental factors that contribute to chemo-resistance [4, 46]. Most solid tumors develop hypoxic areas as a result from abnormal vesicular formation. The mechanisms by which hypoxia induces chemo-resistance remain poorly understood. The hypoxia responsive transcription factor HIF-1α has been associated with chemotherapy failure [47–49]. Chemically or genetically inhibition of HIF-1α prevents chemo-resistance in many cancer cells *in vitro* and *in vivo* [50–55]. Our studies find that Nrf2 directly regulates HIF-1α expression under hypoxia conditions to promote cisplatin-resistance in hepatocellular carcinoma cells, providing novel knowledge on drug resistance in the hypoxic tumor micro-environment.

In the process to evaluate the effect of hypoxia on cisplatin resistance in hepatocellular carcinoma cells, we notice an interesting difference in HIF-1α protein expression patterns: 5% O_2_ transiently increases HIF-1α protein levels in HepG2 cells but constitutively increases in HepG2/DDP cells. This difference is correlated with cisplatin-resistance. HepG2/DDP cells have elevated Nrf2 activity, which contributes to drug resistant phenotypes [39]. Following this clue, we study the roles of Nrf2 in hypoxia-induced cisplatin resistance and find that Nrf2 binds directly to an enhancer element in the upstream of HIF-1α gene and promotes its expression under hypoxia conditions. By implicating Nrf2 in hypoxia-induced drug resistance and providing evidence for direct interaction of Nrf2 protein with HIF-1α enhancer, our study has gained significant insights into the underlying mechanisms by which the hypoxic tumor micro-environment causes chemo-resistance.

**Figure 5:**
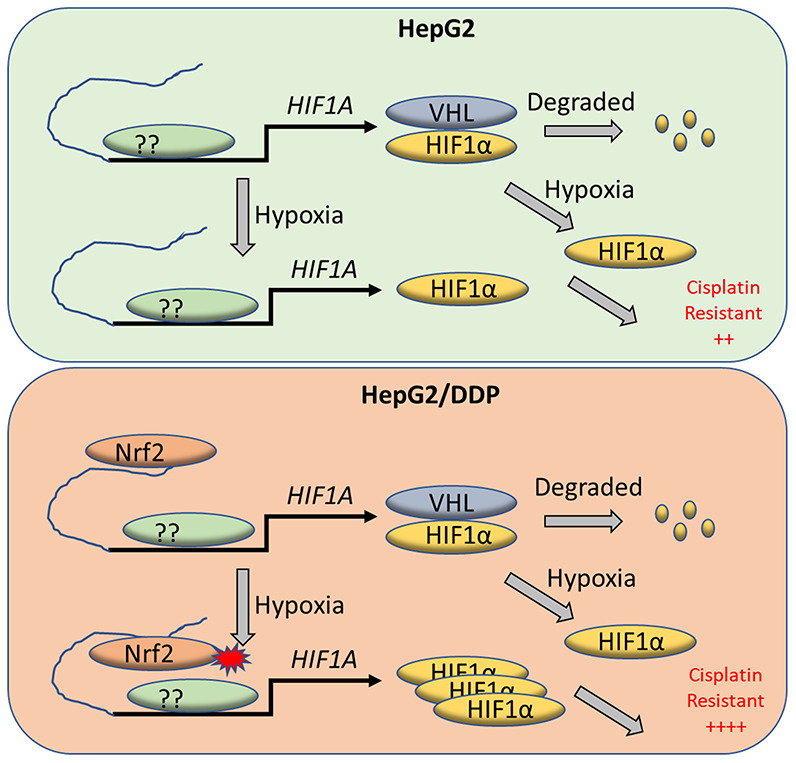
**A working model for Nrf2 regulation of HIF-1α for cisplatin-resistance in HepG2/DDP cells under hypoxia.** Tumor micro-environment is hypoxic, which contributes to cancerous progression and chemo-resistance through complex mechanisms. HIF-1α protein is normally translated then rapidly degraded by proteasome through VHL-mediated ubiquitination. Under hypoxia conditions however, HIF-1α degradation is inhibited and accumulates in the nucleus to induce adaptive transcriptional programs, leading to cisplatin resistance. We find in the cisplatin-resistant hepatocellular carcinoma cell line HepG2/DDP that Nrf2 binds to HIF-1α enhancer. The enhancer-bound Nrf2 serves to augment the transcription of HIF-1α by other transcription factors, leading to increased cisplatin resistance under hypoxic stress.

Interestingly, the binding of Nrf2 to the enhancer element of HIF-1α is not oxygen-sensitive. Regardless of normoxia or hypoxia, stronger Nrf2 binding is found in HepG2/DDP cells compared to HepG2 cells (Figure 4F). Despite the weaker Nrf2 binding, HIF-1α induction remains sensitive to hypoxia in HepG2 cells (Figure 1D–1F). Similarly, Nrf2 hyperactivation does not significantly up-regulate HIF-1α expression under normoxia (Figure 2F, 2G, time 0 of hypoxia). These results suggest that Nrf2 binding to HIF-1α enhancer is not sufficient to promote HIF-1α expression and cisplatin resistance. Instead, the enhancer-bound Nrf2 likely acts to license HIF-1α expression in HepG2/DDP cells (Figure 5). Upon further hypoxic stimulation, the enhancer-bound Nrf2 serves to augment the transcription of HIF-1α by other factors, culminating in sustained induction of HIF-1α in HepG2/DDP as compared to HepG2 cells (Figure 5). This role fits into the traditional function of an enhancer. The transcription factor(s) that cooperates with Nrf2 to regulates HIF-1A gene expression remains to be explored. Our study suggests that mutations in the Nrf2 pathway might also activate similar mechanisms in the hypoxic microenvironment to promote chemo-resistance.

The differential regulation of HIF-1α between HepG2 and HepG2/DDP is restricted to mild hypoxic conditions (5% O_2_). We have also tried 1% and 2%O_2_ but HIF-1α was constitutively induced in both HepG2 and HepG2/DDP cells (data not shown). Traditionally, hypoxia is a relative term and various O2 concentrations have been used in cell culture. Tumor microenvironment is differentially oxygenated, with oxygen supply ranging from ~12% to 1% depending on the distance from blood vessels [56]. Therefore, our observation in 5% O2 concentration should have physiological relevance.

## MATERIALS AND METHODS

### Cell culture and hypoxia

HepG2 and HepG2/DDP cells were obtained from the Cell Bank, Chinese Academy of Sciences. Cells were maintained in RPMI-1640 medium supplemented with 5% fetal bovine serum in a humid incubator with 5% CO_2_ at 37° C. 0.1 ug/ml cisplatin was added to the medium for HepG2/DDP cells. Cells were split every 3-4 days and discarded if passage generation is over 40. To stress cell with hypoxia, cells were incubated in an oxygen-control incubator with a setting of 5% oxygen.

### siRNA knockdown, drug treatment and HIF-1A overexpression

Nrf2, KEAP1 and VHL siRNAs were purchased from Santa Cruz Biotechnology. To knock down genes, cell at 60% confluency were transfected with siRNA with Lipofectamine RNAiMAX Reagent (ThermoFisher)‎, according to manufacturer’s protocol. Cisplatin was dissolved in water in a stock solution of 1mg/ml and add to cell culture to the final concentration of 10ug/ml. Dimethyl-bisphenol A (BPA) was dissolved in DMSO and added to the cell culture to the final concentration of 100uM. pcDNA3 plasmid expressing nondegradable HIF-1α(P402A/P564A) [43] was from Addgene (Constructs #18955). Plasmid were purified from bacteria and transfected into HepG2 cells with Lipofectamine 3000. An empty pcDNA3 plasmid was used as a control.

### Viability assay

Viability was measured by using CellTiter-Glo viability assay reagents from Promega as follow. Cells cultured on 96-well plate were treated with siRNA or drugs as indicated in each experiment. Cell culture media were removed and 50 ul of PBS was added to the cells. Immediately, 50 ul of CellTiter-Glo reagent was added to the cells and cells were lyzed by shaking the plates on an orbital shaker at room temperature for 5 min. Plates were then left in the dark at room temperature for another 10 min then luminescent intensity was read with a plate reader. Shown data were average of at least 3 experiments.

### Real-time quantitative PCR (RT-qPCR)

Total RNA was extracted from cells with TRIzol RNA reagent (ThermoFisher). RNA was reverse transcribed to cDNA using Promega Reverse Transcription System. Real-time PCR was performed using SYBR premix Ex Taq II (Takara) as reported before [57]. Briefly, RT-qPCR was done with 10 pmol of each primer, 100 ng of cDNA, and Go Taq Green Master Mix (Promega, Madison, WI, USA), and the condition was as follows: first 2 min at 94° C and then 30 s at 94° C, 30 s at 55° C, and 30 s at 72° C. The primers for human *HIF-1A* were 5′-CCT AAC GTG TTA TCT GTC GC-3′ and 5′-GTC AGC TGT GGT AAT CCA CT-3′. Primers for human β-actin gene were 5′-CAA GAG ATG GCC ACG GCT GCT-3′ and 5′-TCC TTC TGC ATC CTG TCG GCA-3′. The primers for human *GLUT-1* gene were 5′-TTCACTGTCGTGTCGCTGTTT and 5′-AGCGCGATGGTCATGAGTAT. Shown data were average of at least 3 experiments.

### Western blotting

Cell culture medium was removed and 1X SDS-PAGE loading buffer (62.5 mM Tris-HCl pH 6.8; 2.5 % SDS; 0.002 % Bromophenol Blue; 0.7135 M (5%) β-mercaptoethanol; 10 % glycerol) were added directly to the cells, followed by rapid pipetting 5 times with regular pipette tips. Whole cell lysate was heated at 95 ° C for 5 min then separated by SDS-PAGE. To separate nuclear and cytoplasm, cells were trypsinized and detached form plates, washed with PBS. Cytoplasm and nuclei were then separated using NE-PER™ Nuclear and Cytoplasmic Extraction Kit (Thermo Fisher Scientific) by following manufacture’s protocol. Proteins separated by SDS-PAGE then transferred to PVDF membrane, blocked in 5% non-fat milk then probed with primary antibodies in 5% non-fat milk according to manufacturer’s guideline. Membrane were washed extensively and probed with HRP-conjugated secondary antibody. Membrane was detected by enhanced chemiluminescence (ECL). HIF-1α (ab82832), Lamin B (ab140411) and GAPDH (ab9485) were purchased from Abcam. KEAP1(#30435), Nrf2 (#30597) Actin (#20270) were purchased from Promab Biotechnologies, Richmond CA, USA. Shown data were average of at least 3 experiments.

### Chromatin-immunoprecipitation (ChIP)

Immunoprecipitation of Nrf2 was performed by using ExactaChIP Chromatin Immunoprecipitation Kits (R & D system) and a rabbit anti-Nrf2 polyclonal antibody (ab137550) from Abcam. Cells were fixed with 1% Formaldehyde for 15 min then 125mM Glycine was immediately added to prevented further crosslinking. Cells were collected by centrifugation and lyzed in lysis buffer (within the kit) supplemented with protease inhibitor (10 μg/mL Leupeptin, 10 μg/mL Aprotinin, and 1 mM PMSF). Cells were sonicated to break DNA into an average length of 1Kb and centrifuged at 12,000g for 10 min to collect supernatant. Control IgG or Nrf2 antibody was added and incubated for 30 min, followed by biotinylated secondary antibody for 15 min. magnetic streptavidin beads were added and incubated for 30 min at 4° C, followed by washing with included washing buffer on a magnetic stand. Samples were boiled and subject to DNA purification. RT-qPCR or regular PCR were carried out to detect the presence of the Nrf2 binding site on HIF-1α promoter using the primers set as follow: GCCCTTGGGTGGATGGTGTT and CAACGAAGGGCACTTTCATTA. Shown data were average of at least 3 experiments.
